# Adaptation of the Human Gut Microbiota Metabolic Network During the First Year After Birth

**DOI:** 10.3389/fmicb.2019.00848

**Published:** 2019-04-24

**Authors:** Alvaro Fuertes, Sergio Pérez-Burillo, Iñigo Apaolaza, Yvonne Vallès, M. Pilar Francino, José Ángel Rufián-Henares, Francisco J. Planes

**Affiliations:** ^1^Bioinformatics Group, TECNUN School of Engineering, Department of Biomedical Engineering and Sciences, Universidad de Navarra, San Sebastián, Spain; ^2^Bioinformatics Group, CEIT, Water and Health Division, Universidad de Navarra, San Sebastián, Spain; ^3^Departamento de Nutrición y Bromatología, Instituto de Nutrición y Tecnología de los Alimentos, Centro de Investigación Biomédica, Universidad de Granada, Granada, Spain; ^4^Unitat Mixta d’Investigació en Genòmica i Salut, Fundación para el Fomento de la Investigación Sanitaria y Biomédica de la Comunitat Valenciana-Salud Pública/Instituto de Biología Integrativa de Sistemas, Universitat de València, Valencia, Spain; ^5^Department of Biological and Chemical Sciences, The University of the West Indies, Cave Hill, Barbados; ^6^CIBER en Epidemiología y Salud Pública, Madrid, Spain; ^7^Instituto de Investigación Biosanitaria ibs.GRANADA, Universidad de Granada, Granada, Spain

**Keywords:** personalized nutrition, metabolic networks, human gut microbiome, metagenomics, metabolomics

## Abstract

Predicting the metabolic behavior of the human gut microbiota in different contexts is one of the most promising areas of constraint-based modeling. Recently, we presented a supra-organismal approach to build context-specific metabolic networks of bacterial communities using functional and taxonomic assignments of meta-omics data. In this work, this algorithm is applied to elucidate the metabolic changes induced over the first year after birth in the gut microbiota of a cohort of Spanish infants. We used metagenomics data of fecal samples and nutritional data of 13 infants at five time points. The resulting networks for each time point were analyzed, finding significant alterations once solid food is introduced in the diet. Our work shows that solid food leads to a different pattern of output metabolites that can be potentially released from the gut microbiota to the host. Experimental validation is presented for ferulate, a neuroprotective metabolite involved in the gut-brain axis.

## Introduction

The study of nutrition has become increasingly concerned with human metabolism and the individualized human metabolic responses to diet. This approach was defined as personalized nutrition or nutrigenetics (Mutch et al., 2005). However, although personalized nutrition is frequently considered in the context of diet–gene interactions, individual human physiology depends not only on human genes, but also on the gut microbiota (Sonnenburg and Bäckhed, 2016). The gut harbors a densely populated microbial ecosystem containing a number of bacterial cells larger than the number of eukaryotic cells in the entire human body. The colon is the major site for the gut microbiota’s ‘co-metabolic’ activity, which enhances the efficiency of energy harvest from foods and influences the synthesis, bioavailability, and function of nutrients (Tremaroli and Bäckhed, 2012). This activity produces different beneficial compounds that regulate host health, such as short chain fatty acids (SCFAs), polyphenol metabolites, neuroactive chemical species, etc. In this context, one of the major challenges in nutrition and health is to elucidate the interaction between diet and the metabolism of the gut microbiota (den Besten et al., 2013).

Systems Biology and metabolic networks are an elegant approach to predict the overall functionality of the gut microbiota as well as the biosynthesis of specific health-related metabolites in response to diet. Current network-based methods to analyze gut microbiota metabolism are divided in two different strategies. On the one hand, some studies have used a supra-organism approach, which ignores boundaries for species and models community-level metabolism, based on graph-theory, by integrating metagenomics (Greenblum et al., 2012) or taxonomic data (Sridharan et al., 2014) with metabolic reaction repositories, such as KEGG (Kanehisa et al., 2016) or SEED (Henry et al., 2010). A more evolved approach than graph-based methods is constraint-based modeling (CBM), which includes mass-balance and thermodynamic constraints (Price et al., 2004). Current CBM approaches focus on inter-species models, requiring the genome-scale metabolic reconstruction of each organism as input data. A remarkable work was recently presented in Magnúsdóttir et al. (2017), which released the first large-scale human gut microbiota reconstruction involving 773 different species resident in the human gut. These methods typically integrate 16S rRNA sequence data of bacterial species contained in the samples. Despite these relevant advances, multi-species CBM is still in its infancy and key technical challenges must be addressed (Magnúsdóttir and Thiele, 2018).

In a previous work (Tobalina et al., 2015), we presented a mixed approach that builds on CBM but, at the same time, uses a supra-organismal strategy. In particular, our approach was constructed to identify metabolic networks that capture the differences between two scenarios of interest based on the functional and taxonomic assignments of available meta-omics data. In this work, this algorithm is extended and applied to elucidate the metabolic changes induced over the first year after birth in the gut microbiota of a birth cohort of Spanish infants. To that end, we used metagenomics data of fecal samples and nutritional data for 13 infants at five time points during the first year after birth. Our aim is to analyze the resulting context-specific metabolic networks for each time point considered and establish metabolic differences.

## Materials and Methods

### Reference Metabolic Network

We obtained the list of reactions and metabolites from the Model Seed (Henry et al., 2010), a freely available resource to reconstruct, compare, and analyze genome-scale metabolic networks. We introduced the following changes: first, in order to model compounds that can be potentially released by the gut microbiota to the human host, we added an irreversible output exchange reaction for each metabolite defined in the extracellular compartment of the Model Seed database. Second, we extracted from the metabolic model presented in Heinken and Thiele (2015) the subset of output exchange reactions not considered in the previous step. Overall, we have 717 output metabolites in our reference metabolic network. Third, we defined an irreversible input exchange reaction for each input metabolite identified in our nutritional assessment of infants involved in the study (see below). In total, we have 135 input nutrients, including minerals, carbohydrates, amino acids, vitamins, lipids, fiber, and flavonoids. Fourth, as in Tobalina et al. (2015), we included a biomass reaction in our network, which represents a consensus equation for the metabolic requirements of different members of the human gut microbiota to support growth. In total, we have 17664 metabolites and 14124 reactions, which are stored in the stoichiometric matrix, S.

Our objective is to contextualize this reference metabolic network for each condition in our study based on available metagenomics and nutritional data. In other words, we aim to select a particular subset of active metabolites and reactions for each condition. To that end, the algorithm presented in Tobalina et al. (2015) was applied. As noted above, this algorithm uses a supra-organismal strategy to select active reactions; however, unlike existing graph-based methods, the resulting context-specific metabolic networks satisfy mass-balance constraints and biomass production, as typically done in CBM. On the other hand, although this algorithm was first tested with metaproteomics data, it can be similarly used in cases where metagenomics or metatranscriptomics data are available. Clearly, metaproteomics data are more reliable to infer active enzymes in a microbial community; however, metagenomics is more common in the literature and widely used to infer metabolic capabilities (Greenblum et al., 2012; Sridharan et al., 2014), as it is done here. In addition, although the correlation between gene abundances and mRNA/protein levels has not been sufficiently explored, it is considerably high in some cases reported in the literature (Franzosa et al., 2014; Zhao et al., 2015), which supports the analysis conducted here. We describe below how metagenomic and nutritional data were integrated into the algorithm in Tobalina et al. (2015).

### Metagenomic Data

From Vallès et al. (2014), we collected 454 pyrosequencing metagenomic data of the gut microbiota of 13 Spanish infants at five different time points during the first year after birth (1 week and 1, 3, 7 months, and 1 year). For the second time point considered (1 month), we only have data for 9 out of 13 infants and, therefore, we have 61 samples overall. Information on sex, type of delivery, antibiotic exposure, and feeding habits for these infants is provided in Supplementary Table S1. In brief, all infants were born at term (>37 weeks of gestation), 10 of them by vaginal delivery and three by C-section. Their mothers had not taken antibiotics in at least 3 months before the onset of labor. Six women received antibiotics during delivery. Nine infants were exclusively breastfed during at least 3 months, three received a few formula feedings during the first days of life and one was partially breastfed during the first month and formula-fed thereafter. All infants remained healthy throughout most of the sampling period and solid foods were introduced into their diets between 4 and 6 months after birth, following typical patterns of Spanish Mediterranean infant diets. Previous statistical analyses have established that metagenomic variation in these samples is mainly driven by the infants’ age, as differences among infants within a sampling time point (including those that may result from variation in mode of birth, feeding regime or antibiotic use) are smaller than those present among infants of different ages (Vallès et al., 2014). This justifies the comparisons among metabolic networks at different time points presented in Section “Results.”

The functional annotation of sequenced reads was conducted using HMMER2 (Finn et al., 2011) against TIGRFAMs database 9.0 (Haft et al., 2003). As a result, for each of the 61 samples available, we obtained the read count assigned to 2703 proteins annotated in TIGRFAMS (Supplementary Data S1). We denote a_ijt_ the read count for protein *i* (*i = 1,…,N*) in infant *j* (*j = 1,…,J*) at time point *t* (*t = 1,…,T*). *N*, *J*, and *T* are the total number of TIGRFAMS proteins, infants and time points, respectively.

On the other hand, the taxonomic assignment of sequenced reads was carried out with BLASTX (Altschul et al., 1990), obtaining for each analyzed sample the read count for 632 taxa (Supplementary Data S1). We denote b_wit_ the read count for taxonomy *w* (*i = 1,…,W*) in infant *j* (*j = 1,…,J*) at time point *t* (*t = 1,…,T*). *W* is the total number of taxonomies considered.

#### Absolute Classification of TIGRFAMs Proteins

Functional metagenomics data were first summarized per time period, namely $a_{it}=\sum_{j=t}^{J}a_{ijt}$, which substantially reduces the variability in sequencing depth for different samples and increases the read count data for the cases analyzed. For each time point considered, we identified the subset of highly (*H_t_*) and lowly (*L_t_*) abundant (TIGRFAMs) proteins based on summarized read counts. To that end, we take as a null hypothesis that all proteins are equally abundant and, therefore, assume that the read count for each protein follows a Poisson distribution ${\text{χ}}_{it}({\hat{\text{λ}}}_{t})$, where the mean value is normalized by time point: ${\hat{\text{λ}}}_{t}=\sum_{i=1}^{N}a_{it}/N$. We consider as lowly abundant those proteins with an observed read count significantly less abundant than expected under the above hypothesis (significant threshold: *p*-value ≤ 0.05; *p*-value = $p({\text{χ}}_{it}({\hat{\text{λ}}}_{t})\leq a_{it}))$. If the opposite occurs, we consider such protein as highly abundant.

#### Differential Classification of TIGRFAMs Proteins

In order to avoid the selection of lowly abundant proteins, we first filtered proteins (i) that were classified as lowly expressed in all time points considered or (ii) for which 50% of infants had no reads assigned in all time points considered. We then conducted differential abundance analysis for the rest of proteins in the TIGRFAMs database between each successive time point. This analysis was done with *edgeR* (Robinson et al., 2009), using the trimmed mean of *M*-values (TMM) normalization, which blocks different sources of variability associated with read count data. We selected as differentially abundant proteins between two successive time points (*K_t,t+1_*) those proteins with *p*-value ≤ 0.05. Again, we removed from *K_t,t+1_* those proteins classified as lowly expressed in time points *t* and *t+1* or proteins for which 50% of infants had no reads assigned in time points *t* and *t+1*.

#### Taxonomic Analysis

Again, in order to reduce the sequencing depth variability among samples, we first summarized the taxonomic assignment per time period, namely $b_{wt}=\sum_{j=1}^{J}b_{wjt}$. Second, for each time period, we selected those taxa with an abundance (*x_t_*) higher than 1%: $x_{t}\;=\;\{w\text{∣}\;(b_{wt}/\sum_{w=1}^{W}b_{wt})\geq 0.01\}$, as done in Vallès et al. (2014). For these taxa, we obtained the set of related genomes from the KEGG website (Kanehisa et al., 2016). Enzymes from these genome annotations that were neither included in proteins in *H_t_* nor *L_t_* were included in *M_t_*. Full details regarding these taxa and genome annotations can be found in Supplementary Data S1.

#### Summary

Based on metagenomic data, for each time period considered, we have a different set of highly abundant (*H_t_*) and lowly abundant (*L_t_*) TIGRFAMs proteins, as well as a different set of enzymes annotated from relevant taxonomies (*M_t_*). We denote the set of enzymes from the reference metabolic network not included in *H_t_*, *L_t_*, or *M_t_* as *D_t_*. Namely, *D_t_* involves the subset of non-identified enzymes that are currently annotated for organisms not present in the community. Note here that we used Enzyme Commission (EC) number to code for enzymes. Metabolic proteins annotated in TIGRFAMS have at least one EC number assigned. By linking enzymes to reactions via EC numbers, sets *H_t_*, *L_t_*, *M_t_*, and *D_t_* can be transformed to the reaction level for each time step. The same can be done for *K_t,t+1_*, the list of differentially abundant TIGRFAMs proteins between two consecutive time-steps.

### Nutritional Data

In order to assess the daily intake of food and nutrients for each infant, we used a semi-quantitative food frequency questionnaire based on the validated questionnaire by Vioque et al. (2013). The infants’ food consumption was specified by their mothers. Food frequency consumption of different infants was recorded 1 week and 1, 3, 7, and 12 months after birth, similarly to metagenomic data, taking into account lactation, the formulas used and the regular food for supplementing lactation. Nutrient intake was calculated using the online software i-Diet^1^, which was developed for the use of professionals in the field of nutrition and dietetics. As a result, daily consumption of 135 nutrients was obtained (see Supplementary Data S1).

For each time point, we identified the active input metabolites and added their associated exchanges to *H_t_.* Instead, the exchange reactions associated with inactive input metabolites (zero abundance) were excluded from the reference network. On the other hand, the relative abundance of identified metabolites between each successive time point was compared using a paired *t*-test. We used the following threshold cutoff for differentially abundant metabolites: *p*-value ≤ 0.05 and increase/decrease by fold-change ≥ 1.5. Exchange reactions that are associated with differentially abundant metabolites were included in the set *K_t,t+1_*.

### Data Integration and Metabolic Reconstruction

As detailed in Tobalina et al. (2015), we seek a functional network that includes the maximum number of highly likely reactions (H_t_) and the minimum number of lowly likely reactions (L_t_). We complete the network using the reactions in the reference network, preferably those annotated in taxonomic groups present in the community (M_t_). Note here that, in order to capture the metabolic differences between time points considered, we particularly force the inclusion of the maximum number of over-abundant TIGRFAMs enzymes and input metabolites in each situation.

As typically done in CBM, the selected reactions must satisfy the mass balance equation, the growth medium and thermodynamic constraints and the biomass production:

$$\begin{array}{l} S\cdot v=0\;\;\;\;\;\;\;\;\;\;\;\;\;\;\;\;\;\;\;\;\;\;\;\;\;\;\;\;\;\;\;\;\;\;\;\;\left(1\right) \end{array}$$

$$\begin{array}{l} v^{min}\leq v\leq v^{max}\;\;\;\;\;\;\;\;\;\;\;\;\;\;\left(2\right) \end{array}$$

$$\begin{array}{l} v_{bio}\geq \text{ε}\;\;\;\;\;\;\;\;\;\;\;\;\;\;\;\;\;\;\;\;\;\;\;\;\;\;\;\;\;\;\;\;\;\;\;\;\;\;\left(3\right) \end{array}$$

where *v* represent reaction fluxes, *v^min^* and *v^max^* the lower and upper bounds for reaction fluxes, respectively, *v_bio_* the flux through the biomass reaction and ε the minimum required flux through the biomass reaction. Note here that, aside from input and output reaction exchanges, the rest of reactions are potentially reversible and they are split into two different steps (forward and backward reactions) with non-negative fluxes (*v^min^* = 0). In addition, we fixed *v_j_^max^* = *α* = 1000, except for exchange reactions associated with inactive input metabolites, whose upper bound is zero (equivalent to deletion). Finally, we set ε = 1. In Tobalina et al. (2015), it was shown that the results are robust to the value of ε and *α*.

In order to guide the search of a reaction network that satisfies Eqs (1)–(3) and takes into account metagenomics and nutritional data, we used the algorithm presented in Tobalina et al. (2015), which consists of a three-step iterative procedure based on linear optimization and a reaction scoring based on the classification of reactions described above. In the first two steps (Steps 1–2), steady-state central metabolic pathways for biomass production are established based on single reaction knockout perturbations. Here, we also included double reaction knockout perturbations to have more complete networks. The resulting networks are then expanded to include over-abundant nutrients and enzymes and emphasize metabolic differences at each scenario (Step 3). In order to have a more complete view of the output metabolites obtained from over-abundant nutrients and enzymes, we implemented a single reaction knockout perturbation strategy for output metabolites obtained in Step 3.

A step-by-step description of the algorithm can be found in the Supplementary Methods. The algorithm was implemented in MATLAB, using IBM Ilog Cplex to solve optimization problems. A Matlab implementation of our algorithm is available in the Supplementary Code.

### Metabolomic Validation

To validate our approach (see section “Results”), we measured the levels of ferulate (ferulic acid) in different time steps using a targeted metabolomics approach. Note here that we used aliquots of the same samples from which metagenomic data was obtained. Details are presented below.

#### Extraction of Ferulic Acid

Fecal samples frozen upon collection were processed by resuspension of approximately 200 mg of sample per mL of phosphate buffered saline. Samples were sonicated for 15 min and centrifuged at 13000 rpm for 10 min, and the supernatant was set aside. One mL of the supernatant was mixed with 1 mL of diethyl ether in a 2 mL tube and was kept in the dark for 24 h. Afterward, the supernatant (containing diethyl ether along with phenolic compounds) was separated into a clean 10 mL tube. Thereafter, 1 mL of diethyl ether was added to the 2 mL tube, mixed by inversion and the supernatant (diethyl ether) separated into the 10 mL tube. This step was repeated one more time, so that 3 mL of diethyl ether were collected into the 10 mL tube. Afterward, anhydrous sodium sulfate was added to eliminate humidity. Diethyl ether was then evaporated with vacuum at 30°C. Phenolic compounds were resuspended in 1 mL of a water:methanol mix (50:50) and transferred to a high-performance liquid chromatography (HPLC) vial right after filtering them through a 0.22 μ filter.

#### HPLC Measurement of Ferulic Acid

Ferulic acid identification was carried out by HPLC following the method described in Moreno-Montoro et al. (2015). The HPLC system was a Thermo Fisher-Scientific Accela 600 equipped with a quaternary pump, an autosampler, a column oven and a variable wavelength UV-vis detector (PDA) set at 280 nm. The analytical column was a reverse phase C18 column thermostatized at 25°C. Mobile phase A was water with 0.1% of formic acid and phase B was acetonitrile with 0.1% of formic acid. The method was carried out with a flow rate of 0.7 mL/min with the following gradient: 0% of B for 15 min, 100% of B at minute 110, 100% of B for 10 min and 0% of B for 5 min. Twenty μL were injected and the ferulic acid peak was identified by comparison with a reference standard. A calibration curve was performed with a reference standard with concentrations ranging from 5 to 0.0001 ppm.

#### Cell Count

To ensure that measurements were obtained for similar amounts of bacterial cells, we evaluated the number of cells per mL in each sample suspension to determine the volume required for the ferulic acid assay. Cell count was performed with a Neubauer Haemocytometry chamber, which is the standard procedure for cell counting. Cells were resuspended in 1 mL of water and diluted accordingly to obtain around 100 cells per large square in the hemocytometer. Trypan blue was added to this suspension to dye cells and facilitate counting. Ten μL of such suspension were placed in the hemocytometer and cells were counted in the four corners of the 5x5 grid, obtaining afterward an average value for the four corners.

## Results

Based on Tobalina et al. (2015) and data presented above, we calculated a consensus metabolic network for the gut microbiota of infants of 1 week and 1, 3, 7, and 12 months of age. It is important to note that these networks are not fully comprehensive but they emphasize the main metabolic differences across two consecutive conditions. Full details as to the reactions involved for each case can be found in Supplementary Data S1.

**FIGURE 1:**
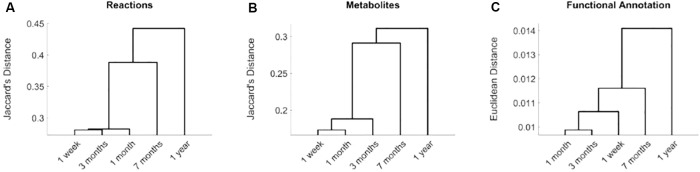
Hierarchical clustering analysis of reconstructed metabolic networks of gut microbiota of infants at 1 week and 1, 3, 7 months, and 1 year of age. Distances based on active reactions **(A)**, metabolites **(B)** and functional metagenomic annotations **(C)**. In **(A)**, for each time point, we defined a binary vector that stores active reactions in its reconstruction. We compared these binary vectors for the different time points using Jaccard’s distance. A similar analysis was done for metabolites in **(B)**. For functional metagenomic annotation data in **(C)**, we used Euclidean distance.

Based on Jaccard’s distance, we evaluated the similarity at the reaction and metabolite levels between the different computed networks (Supplementary Table S2) and conducted hierarchical clustering analysis (Figure 1A,B). Networks associated with data collected after 7 months and 1 year of birth are clearly separated from data taken after 1 week and 1 and 3 months. This significant change is related with the introduction of solid food, between 4 and 6 months after birth, which modifies nutritional patterns and, thus, the input exchange reactions (active nutrients) in the reconstructed networks. The effect of solid diet is more clearly observed after 1 year, where we found more significant differences at both taxonomic and functional level, as discussed in detail in Vallès et al. (2014). This analysis shows that we were able to capture the main metabolic network adaptation during the first year after birth. Note here that the computed metabolic networks capture more clearly the effect of solid foods than functionally annotated metagenomic data, as observed in the dendrogram of Figure 1C, which reinforces the usefulness of the integrative approach presented here.

For every pair of successive time points, we compared the metabolic pathways involved in their resulting networks through KEGG maps (see Supplementary Tables S3–S6). However, in order to summarize the functional changes associated with the introduction of solid diet, following the results in Figure 1, we merged the metabolic networks before (1 week and 1 and 3 months) and after (7 months and 1 year) the solid diet introduction and analyzed KEGG maps. For comparing both scenarios, we used a dissimilarity score (*J_p_*), based on Jaccard’s distance, which was introduced in Tobalina et al. (2015). We ranked the KEGG pathways according to this measure (see Supplementary Data S1).

Table 1 shows the top 10 most dissimilar KEGG pathways between metabolic networks before and after solid food introduction. The importance of the metabolism of phenolic compounds after the introduction of solid food is clearly reflected in Table 1 with the activation of “Flavonoid biosynthesis” and “Phenylpropanoid biosynthesis” maps. These changes are linked to the intake of fruits and vegetables. In addition, infants before solid diet introduction seem more dependent on vitamin B6 metabolism, which is in line with previous reports suggesting the need for supplementation during breast-feeding (Falsaperla et al., 2017).

**Table 1:** Ten most dissimilar KEGG pathways between metabolic networks before and after solid food introduction.

KEGGID	Name	Before solid diet	After solid diet	Dissimilarity score
map00941	Flavonoid biosynthesis	0	18	18.00
map00630	Glyoxylate and dicarboxylate metabolism	27	21	7.65
map00130	Ubiquinone and other terpenoid-quinone biosynthesis	6	16	6.25
map00940	Phenylpropanoid biosynthesis	1	8	6.13
map00622	Xylene degradation	1	7	5.14
map00523	Polyketide sugar unit biosynthesis	0	5	5.00
map00750	Vitamin B6 metabolism	4	1	4.00
map00360	Phenylalanine metabolism	11	7	3.69
map00500	Starch and sucrose metabolism	14	15	3.30
map00440	Phosphonate and phosphinate metabolism	0	3	3.00


*For each KEGG pathway in table, the entries in “Before solid diet” and “After solid diet” columns represent the number of its annotated reactions that are involved in the merged reconstructions before and after solid diet introduction, respectively.*

Importantly, the modulation of the gut microbiota of infants after the introduction of solid diet leads to different output metabolites (Supplementary Data S1). These output metabolites may be released to human cells and fluids and, thus, regulate host health. Among the predicted output metabolites that differentiate the networks before and after solid food introduction, we focused on ferulate (ferulic acid), which is a phenolic compound involved in the “Phenylpropanoid biosynthesis” KEGG map. Ferulate is a neuroprotective metabolite (Cheng et al., 2008), involved in the gut-brain axis (Westfall et al., 2017), which has been previously associated with cognitive development in embryonic rats (Yabe et al., 2010).

**FIGURE 2:**
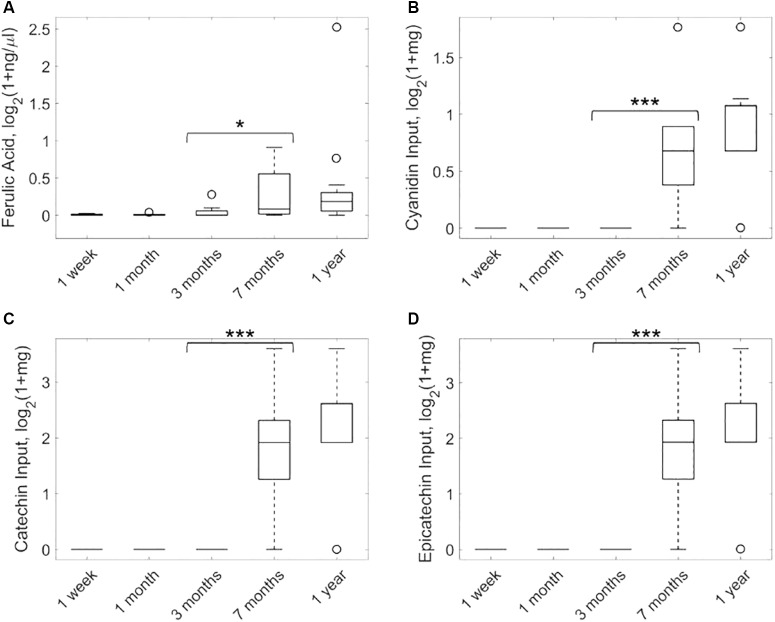
Analysis of ferulate production in feces samples taken from infants over the first year after birth. Metabolomic analysis of ferulate **(A)** and consumption of cyanidin **(B)**, catechin **(C)**, and epicatechin **(D)** (precursors of ferulate) based on nutritional data. ^∗^*p* < 0.05, ^∗∗∗^*p* < 0.001.

In order to evaluate the statistical significance of ferulate, we first conducted 50 bootstrap random permutations of metagenomic and nutritional data and applied our network reconstruction pipeline to each of them. The output exchange reaction of ferulate was active in less than 5% of these random reconstructions, which provides additional support for the result presented here. Figure 2A shows the targeted metabolomic analysis of ferulate in fecal samples during the first year after birth of the infants considered. It can be observed that the levels of ferulate significantly increase after 7 months (one-tailed paired Wilcoxon test, *p*-value = 0.0116), maintaining a similar value after 1 year (non-significant differences between 7 months and 1 year and significant differences between 1 year and the rest of time points). Therefore, the levels of ferulate seem to be linked to solid diet introduction, as predicted by our algorithm.

Based on our metabolic reconstructions, we calculated input nutrients that are degraded to form ferulate. For this analysis, we adapted the K-shortest Elementary Flux Modes algorithm (de Figueiredo et al., 2009) and enumerated minimal combinations of nutrients that produce ferulate (see Supplementary Methods). Three input nutrients were identified: cyanidin, catechin, and epicatechin. Figure 2B shows the consumption of cyanidin of infants in our study along the first year after birth. These data were taken from the available nutritional data described above. It can be observed that the consumption of cyanidin is solid-diet specific, mainly associated with the intake of fruits. A similar result was found for catechin and epicatechin (Figure 2C,D).

Our hypothesis is that, once solid diet is introduced, ferulate starts being synthesized by the gut microbiota of infants from available cyanidin, catechin, and epicatechin. Of course, it may happen that the ferulate found in many plant-based foods (not accounted for in our nutritional data) explains the differences observed in Figure 2A. However, we have found extensive literature supporting that the biosynthesis of ferulate from cyanidin, catechin, and epicatechin is carried out by the gut microbiota (de Ferrars et al., 2014; Yang et al., 2014).

## Discussion

Constraint-based modeling is a promising tool to analyze the interaction of diet, gut microbiota and host. While inter-species metabolic models are currently under development, in this work we apply a supra-organism CBM approach, previously presented in Tobalina et al. (2015), in order to elucidate metabolic changes induced in the gut microbiota of infants during the first year of life based on functional and taxonomic assignment of metagenomics and on nutritional data. Our approach was successful in predicting clear metabolic patterns before (e.g., vitamin B6 metabolism) and after solid foods were introduced (e.g., metabolism of phenolic compounds).

The main application of our approach is to predict active gut microbiota metabolites that could regulate host health, as illustrated in the case of ferulate. In particular, we predict that ferulate starts getting produced in the gut microbiota once solid food is introduced in the infant diet, which is supported by the metabolomic analysis provided and previous literature reporting its biosynthesis from predicted nutrients (cyanidin, catechin, and epicatechin). This result is of interest, since ferulate has been associated with neuroprotection and cognitive development, which reinforces the need and importance of solid food for the infant’s growth.

According to the World Health Organization (WHO) and Food and Agriculture Organization of the United Nations (FAO), complementary feeding should start at the age of 6 months, a time at which the brain and the gut are still developing and maturing. The transition from exclusive breastfeeding to family foods should cover the period from 6 to 18–24 months of age. Both FAO and WHO agree that this period of life is especially important since it is a time of vulnerability and therefore the choice of complementary foods is crucial for the proper physical and neurological development of children. Much work will be needed to understand the impact of solid food introduction patterns on gut microbiota metabolism and infant development and health, but our work demonstrates that the analysis of supra-organismal metabolic networks via CBM methods can help in this endeavor.

## Ethics Statement

This study was approved by the Ethics Committee of the Center for Public Health Research (CSISP), Valencia, Spain. All women participating in the study read and signed forms of informed consent specifically approved for this project by the Ethics Committee.

## Author Contributions

MF, JR-H, and FP conceived this study. IA and AF carried out the computational implementation. SP-B performed the metabolomic experiments. YV generated and processed metagenomic data and collected food frequency questionnaires. All authors wrote, read, and approved the manuscript.

## Conflict of Interest Statement

The authors declare that the research was conducted in the absence of any commercial or financial relationships that could be construed as a potential conflict of interest.

## Footnotes

1 https://i-diet.es/
