# Supplementary material for: Adaptation of the Human Gut Microbiota Metabolic Network During the First Year After Birth
Source: Front Microbiol. 2019 Apr 24;10:848. doi: 10.3389/fmicb.2019.00848 (PMC6491881; doi:10.3389/fmicb.2019.00848)
Supplement: Supplementary file 3 [file Data_Sheet_2.docx]

**Adaptation of the human gut microbiota metabolic network during the first year after birth**

# Supplementary Methods

Assume we have a reference metabolic network that is mathematically represented by the stoichiometric matrix, **S**, where rows are associated with metabolites and columns with reactions. Our objective is to select a particular subset of reactions and metabolites for each scenario based on available metagenomics and nutritional data. The selected reactions must satisfy the mass balance equation, growth medium constraints and biomass production:

$S\cdot v=0$ **(Eq. 1)**

$v^{min}\leq v\leq v^{max}$**(Eq. 2)**

$v_{bio}\geq\varepsilon$ **(Eq. 3)**

In these equations, *v* represent reaction fluxes, *v^min^* and *v^max^* are the lower and upper bounds for reaction fluxes, *v_bio_* the flux through the biomass reaction and $\varepsilon$ the minimum required flux through the biomass reaction. Aside from reaction exchanges, the rest of reactions are potentially reversible and they are split into two different steps (forward and backward reactions) with non-negative fluxes (*v_min_*=0). We allow the inclusion of the backward step of irreversible reactions for gap filling but it is penalized (see below). In addition, we fixed *v_j_^max^*=*α*=1000, except for exchange reactions associated with inactive input metabolites, for which it is zero. Finally, we set $\varepsilon$=1*.* In Tobalina et al., 2015 we showed that the results are robust to the value of $\varepsilon$ and *α*.

**Step 1: Basic Functional Network**

In order to identify a sub-network that satisfies Eqs. (1)-(3) and makes use of metagenomics and nutritional data, we rely on linear programming techniques and define the following objective function:

$min\sum_{j\in R} (p_{j}-b_{j})\cdot v_{j}$ **(Eq. 4)**

, where the penalty (*p_i_*) and bonus (*b_i_*) terms depend on the categorization of reactions. Note here that Eq. (4) makes use of continuous fluxes, which requires non-negativity constraints, since otherwise Eq. (4) is not effective. For this reason, as mentioned above, we need to split reactions into two irreversible steps.

As noted in the main paper, for each condition, we have a different set of highly (*H*) and lowly (*L*) likely reactions, as well as a different set of reactions annotated from relevant taxonomies (*M*). The set of reactions from the reference metabolic network not included in *H*, *L*, or *M* is stored in *D*.

Overall, the weights for fluxes in Eq. (4) are roughly as follows: (*p_i_*-*b_i_*)≈1 for reactions in *H*; (*p_i_*-*b_i_*)≈*10* for reactions in *M*; (*p_i_*-*b_i_*)≈100 for reactions in *D*; (*p_i_*-*b_i_*)≈1000 for backward irreversible reactions; and (*p_i_*-*b_i_*)≈10000 for reactions in *L*. For further details, see Tobalina et al., 2015.

**Step 2: Alternative pathways for biomass production**

Once Step 1 is solved, we obtain a list of active reactions, *N_1_*. In this second step, we aim to extract alternative pathways for biomass production that are not included in *N_1_* using the reactions in *H*, but not in *L*. To this end, we block each reaction *j* in *N_1_*, one-by-one, and resolve the linear program posed in Step 1, *i.e.* Eqs. (1)–(4) (**single reaction knockout analysis**). As a result, we obtain a new functional network for each reaction *j*, *N_2_(j)*. The rule here is to merge *N_2_(j)* with *N_1_* if it includes additional reactions in *H*, but not in *L*. If this is the case, for each new reaction *k* in *N_2_(j),* we repeat the process by blocking simultaneously reactions *j* and *k* (**double reaction knockout analysis**). As a result, we obtain a new functional network for each pair of reactions, *N_2_(j,k)*. Again, the rule is to merge *N_2_(j,k)* with *N_2_(j)* if it includes additional reactions in *H*, but not in *L.* As a result, we obtain a functional network (*N_2_*) that makes better use of the metagenomics data for biomass production. Note here that double reaction knockout analysis was not included in Tobalina et al., 2015.

**Step 3: Network expansion**

In this step, we aim to include the maximum number of differentially abundant TIGRFAMs enzymes and input metabolites (set *K*). To that end, we start for Eqs. (1)-(3). Then, for each reaction *j*, we introduce binary variables *z_j_*, which takes 1 if its associated reaction is active (*v_j_>0*), 0 otherwise. This is ensured with the following constraints:

$z\leq v\leq M\cdot z$**(Eq. 5)**

$z_{f}+ z_{b} \leq1, \forall(f,b)\in B$**(Eq. 6)**

, where *B*={( *f* , *b* )| reaction *f* and reaction *b* are the reverse of each other, *f* < *b*}.

For each enzyme or input metabolite *i* in *K*, we introduce the *e* variables, namely if e=0, we force its inclusion in the reconstruction through the following constraints and objective function:

$0\leq e\leq1$**(Eq. 7)**

$\sum_{j=1, {j\in R}^{i}}^{n} z_{j}+e_{i} \geq1, \forall i\in K$**(Eq. 8)**

$min\sum_{j\backslash N_{2}} (p_{j}-b_{j})\cdot v_{j}+\sum_{i\in K} w_{i}\cdot e_{i}$ **(Eq. 9)**

, where *R^i^* denotes the set of reactions associated with differentially abundant enzymes or input metabolites and *w_i_* is the maximum overall penalty.

Once Eqs. (1)-(3), (6)-(9) are solved, we add a list of active reactions to *N_2_*, leading to *N_3_*. In order to have a more complete view of active output metabolites, we block output exchange reactions *j* in *N_3_* not in *N_2_*, one-by-one, and resolve the optimization model posed in Step 3, *i.e.* (1)-(3), (6)-(9) (**single reaction knockout analysis for output metabolites**). As a result, we obtain a new functional network for each output metabolite, *N_4_(j)*. The rule here is to merge *N_4_(j)* with *N_3_* if it includes additional reactions in *H*, but not in *L*. As a result, we have the final metabolic network *N_4_*, which is the one used in the main manuscript.

**Adaptation of K-shortest EFMs algorithm**

Assume we are interested in finding the minimal number of nutrients required to activate a particular reaction *i* in a metabolic network, which is defined by its stoichiometric matrix, S. To do this, we fist need to satisfy the mass balance equation and irreversibility constraints, and force the reaction *i* to be active:

$S\cdot v=0$ **(Eq. 10)**

$v\geq0$ **(Eq. 11)**

$v_{i}\geq1$ **(Eq. 12)**

Then, we introduce binary variables *z* exclusively for input exchange reactions (**I**), namely if *z_j_=1* then *v_j_≥1* and if *z_j_=0* then *v_j_=0*, which is guaranteed with Eq. (3).

$z_{j}\leq v_{j}\leq M\cdot z_{j} \boldsymbol{\forall j\in I}$ **(Eq. 13)**

Then, by minimizing the number of active *z* variables, we extract the minimum number of input exchange reactions required to support the activity of reaction *i*:

$\min\sum_{j=1, j\in I}^{R} z_{j}$ **(Eq. 14)**

Assume that the solution to Eqs. (10)-(14) is *W*. We denote *|W|* the number of active input exchange reactions in the optimal solution *W*. We can calculate the second shortest solution by adding the following constraint:

$\sum_{j=1, j\in I}^{R} {w_{j}\cdot z}_{j}\leq\left| W \right|\boldsymbol{-1}$ **(Eq. 15)**

This constraint can be repeated as many times as desired (*K*) and obtain the *K* shortest solutions for the question above.

This formulation was adapted from the original one described in de Figueiredo et al., 2009. In this early work, a general method to enumerate minimal number of reactions required to activate a user-defined reaction was presented. In the formulation above, we only considered a subset of the reactions in the minimization function, namely the subset of input exchange reactions instead of the total set of reactions. This algorithm was applied to calculate the source nutrients producing ferulate, as described in the main text.

# Supplementary Tables

**Table S1**: Infants’ information obtained from questionnaires answered by parents.

| **Sample** | **Age** | **Sex** | **Delivery** | **Antibiotics** | **Diet**^a^ |
| --- | --- | --- | --- | --- | --- |
| **MIP01-I1** | 1 week | M | Vaginal, no antibiotics | - | Breast Milk |
| **MIP01-I2** | 1 month | - | - | - | Breast Milk |
| **MIP01-I3** | 3 months | - | - | - | Breast Milk |
| **MIP01-I4** | 7 months | - | - | - | Solids (5 months) |
| **MIP01-I5** | 1 year | - | - | - | Solids |
| **MIP02-I1** | 1 week | F | Vaginal, no antibiotics | - | Mixed |
| **MIP02-I2** | 1 month | - | - | - | Breast Milk |
| **MIP02-I3** | 3 months | - | - | - | Breast Milk |
| **MIP02-I4** | 7 months | - | - | - | Solids (6 months) |
| **MIP02-I5** | 1 year | - | - | - | Solids |
| **MIP03-I1** | 1 week | F | Vaginal, no antibiotics | Oftalmowell^b^ | Breast Milk |
| **MIP03-I2** | 1 month | - | - | - | Breast Milk |
| **MIP03-I3** | 3 months | - | - | - | Breast Milk |
| **MIP03-I4** | 7 months | - | - | - | Solids (5 months 7 days) |
| **MIP03-I5** | 1 year | - | - | Cefuroxime | Solids |
| **MIP06-I1** | 1week | F | C-section, amoxicillin | - | Breast Milk |
| **MIP06-I2** | 1 month | - | - | - | Breast Milk |
| **MIP06-I3** | 3 months | - | - | - | Breast Milk |
| **MIP06-I4** | 7 months | - | - | - | Solids (5 months 12 days) |
| **MIP06-I5** | 1 year | - | - | Amoxicillin | Solids |
| **MIP07-I1** | 1 week | M | C-section, amoxicillin | - | Breast Milk |
| **MIP07-I3** | 3 months | - | - | - | Breast Milk |
| **MIP07-I4** | 7 months | - | - | - | Solids (5 months 23 days) |
| **MIP07-I5** | 1 year | - | - | - | Solids |
| **MIP08-I1** | 1 week | F | Vaginal, no antibiotics | - | Breast Milk |
| **MIP08-I2** | 1 month | - | - | - | Breast Milk |
| **MIP08-I3** | 3 months | - | - | - | Breast Milk |
| **MIP08-I4** | 7 months | - | - | - | Solids (5 months 5 days) |
| **MIP08-I5** | 1 year | - | - | - | Solids |
| **MIP09-I1** | 1 week | M | Vaginal, no antibiotics | - | Mixed |
| **MIP09-I2** | 1 month | - | - | - | Mixed |
| **MIP09-I3** | 3 months | - | - | - | Formula |
| **MIP09-I4** | 7 months | - | - | - | Solids (4 months) |
| **MIP09-I5** | 1 year | - | - | - | Solids |
| **MIP12-I1** | 1week | F | C-section, amoxicillin | - | Mixed |
| **MIP12-I2** | 1 month | - | - | - | Breast Milk |
| **MIP12-I3** | 3 months | - | - | - | Breast Milk |
| **MIP12-I4** | 7 months | - | - | - | Solids (6 months) |
| **MIP12-I5** | 1 year | - | - | - | Solids |
| **MIP13-I1** | 1 week | M | Vaginal, benzylpenicillin | - | Mixed |
| **MIP13-I3** | 3 months | - | - | - | Breast Milk |
| **MIP13-I4** | 7 months | - | - | - | Solids (5 months 6 days) |
| **MIP13-I5** | 1 year | - | - | - | Solids |
| **MIP16-I1** | 1 week | M | Vaginal, amoxicillin | - | Breast Milk |
| **MIP16-I3** | 3 months | - | - | - | Breast Milk |
| **MIP16-I4** | 7 months | - | - | - | Solids (6 months) |
| **MIP16-I5** | 1 year | - | - | - | Solids |
| **MIP17-I1** | 1 week | M | Vaginal, no antibiotics | - | Breast Milk |
| **MIP17-I3** | 3 months | - | - | - | Breast Milk |
| **MIP17-I4** | 7 months | - | - | - | Solids (5 months) |
| **MIP17-I5** | 1 year | - | - | - | Solids |
| **MIP19-I1** | 1 week | F | Vaginal, no antibiotics | - | Breast Milk |
| **MIP19-I3** | 3 months | - | - | - | Breast Milk |
| **MIP19-I4** | 7 months | - | - | - | Solids (4 months 2 days) |
| **MIP19-I5** | 1 year | - | - | - | Solids |
| **MIP21-I1** | 1 week | M | Vaginal, amoxicillin | - | Breast Milk |
| **MIP21-I2** | 1 month | - | - | - | Breast Milk |
| **MIP21-I3** | 3 months | - | - | - | Breast Milk |
| **MIP21-I4** | 7 months | - | - | - | Solids (6 months) |
| **MIP21-I5** | 1 year | - | - | - | Solids |

MIP, Mother Infant Pair. Infant samples collected at one week (I1), one month (I2), three months (I3, before introduction of solid foods), seven months (I4, after introduction of solid foods) and one year after birth (I5).

^a^ The time of solid food introduction for each infant is reported in parentheses in the I4 row.

^b^ Oftalmowell is an eye drops solution containing a combination of gramicidin, neomycin and polymyxin B.

**Table S2**: Jaccard’s distance between reconstructed networks at different time points

|  | **1 week** | **1 month** | **3 months** | **7 months** | **1 year** |
| --- | --- | --- | --- | --- | --- |
| **1 week** | 0.000 | 0.264 | 0.312 | 0.348 | 0.450 |
| **1 month** | 0.264 | 0.000 | 0.315 | 0.391 | 0.455 |
| **3 months** | 0.312 | 0.315 | 0.000 | 0.325 | 0.447 |
| **7 months** | 0.348 | 0.391 | 0.325 | 0.000 | 0.427 |
| **1 year** | 0.450 | 0.455 | 0.447 | 0.427 | 0.000 |

**Table S3:** Ten most dissimilar KEGG pathways between metabolic networks at 1 week and 1 month.

| **KEGGID** | **Name** | **1 week** | **1 month** | **Dissimilarity score** |
| --- | --- | --- | --- | --- |
| map00630 | Glyoxylate and dicarboxylate metabolism | 10 | 19 | 6.81 |
| map00240 | Pyrimidine metabolism | 40 | 41 | 6.13 |
| map00330 | Arginine and proline metabolism | 17 | 24 | 5.89 |
| map00360 | Phenylalanine metabolism | 3 | 11 | 5.81 |
| map00565 | Ether lipid metabolism | 0 | 4 | 4 |
| map00010 | Glycolysis / Gluconeogenesis | 9 | 16 | 3.06 |
| map00627 | Aminobenzoate degradation | 3 | 6 | 2.86 |
| map00910 | Nitrogen metabolism | 9 | 14 | 2.8 |
| map00643 | Styrene degradation | 2 | 6 | 2.66 |
| map00030 | Pentose phosphate pathway | 18 | 15 | 2.57 |

**Table S4:** Ten most dissimilar KEGG pathways between metabolic networks at 1 month and 3 months.

| **KEGGID** | **Name** | **1 month** | **3 months** | **Dissimilarity score** |
| --- | --- | --- | --- | --- |
| map00240 | Pyrimidine metabolism | 41 | 47 | 7.30 |
| map00630 | Glyoxylate and dicarboxylate metabolism | 19 | 15 | 6.40 |
| map00340 | Histidine metabolism | 12 | 9 | 6.12 |
| map00052 | Galactose metabolism | 4 | 11 | 4.45 |
| map00750 | Vitamin B6 metabolism | 0 | 4 | 4.00 |
| map00360 | Phenylalanine metabolism | 11 | 5 | 3.27 |
| map00900 | Terpenoid backbone biosynthesis | 15 | 8 | 3.27 |
| map00643 | Styrene degradation | 6 | 2 | 2.67 |
| map00350 | Tyrosine metabolism | 4 | 4 | 2.57 |
| map00860 | Porphyrin and chlorophyll metabolism | 12 | 17 | 2.33 |

**Table S5:** Ten most dissimilar KEGG pathways between metabolic networks at 3 months and 7 months.

| **KEGGID** | **Name** | **3 months** | **7 months** | **Dissimilarity score** |
| --- | --- | --- | --- | --- |
| map00941 | Flavonoid biosynthesis | 0 | 17 | 17.00 |
| map00340 | Histidine metabolism | 9 | 13 | 8.42 |
| map00564 | Glycerophospholipid metabolism | 25 | 14 | 6.00 |
| map00622 | Xylene degradation | 1 | 7 | 5.14 |
| map00523 | Polyketide sugar unit biosynthesis | 0 | 5 | 5.00 |
| map00600 | Sphingolipid metabolism | 8 | 2 | 4.50 |
| map00750 | Vitamin B6 metabolism | 4 | 0 | 4.00 |
| map00940 | Phenylpropanoid biosynthesis | 1 | 4 | 4.00 |
| map00630 | Glyoxylate and dicarboxylate metabolism | 15 | 14 | 3.30 |
| map00052 | Galactose metabolism | 11 | 5 | 3.27 |

**Table S6:** Ten most dissimilar KEGG pathways between metabolic networks at 7 months and 1 year.

| **KEGGID** | **Name** | **7 months** | **1 year** | **Dissimilarity score** |
| --- | --- | --- | --- | --- |
| map00071 | Fatty acid metabolism | 29 | 3 | 25.20 |
| map00062 | Fatty acid elongation | 18 | 0 | 18.00 |
| map00281 | Geraniol degradation | 12 | 0 | 12.00 |
| map00941 | Flavonoid biosynthesis | 17 | 4 | 11.67 |
| map00340 | Histidine metabolism | 13 | 10 | 7.11 |
| map00730 | Thiamine metabolism | 6 | 0 | 6.00 |
| map00630 | Glyoxylate and dicarboxylate metabolism | 14 | 13 | 5.71 |
| map00232 | Caffeine metabolism | 12 | 4 | 5.33 |
| map00622 | Xylene degradation | 7 | 1 | 5.14 |
| map00523 | Polyketide sugar unit biosynthesis | 5 | 0 | 5.00 |

**References**

de Figueiredo, L. F., Podhorski, A., Rubio, A., Kaleta, C., Beasley, J. E., Schuster, S., et al. (2009). Computing the shortest elementary flux modes in genome-scale metabolic networks. *Bioinformatics* 25, 3158–3165. doi:10.1093/bioinformatics/btp564.

Tobalina, L., Bargiela, R., Pey, J., Herbst, F. A., Lores, I., Rojo, D., et al. (2015). Context-specific metabolic network reconstruction of a naphthalene-degrading bacterial community guided by metaproteomic data. *Bioinformatics* 31, 1771–1779. doi:10.1093/bioinformatics/btv036.
